# Spectroscopic Analysis of the Binding of Paraquat and Diquat Herbicides to Biosubstrates

**DOI:** 10.3390/ijerph18052412

**Published:** 2021-03-02

**Authors:** Francesca Macii, Rebecca Detti, Francesca Rita Bloise, Stefania Giannarelli, Tarita Biver

**Affiliations:** 1 Department of Chemistry and Industrial Chemistry, University of Pisa, 56124 Pisa, Italy; francesca.macii@dcci.unipi.it (F.M.); rebeccadetti@gmail.com (R.D.); francescarita.bloise@libero.it (F.R.B.); stefania.giannarelli@unipi.it (S.G.); 2 Department of Pharmacy, University of Pisa, 56126 Pisa, Italy

**Keywords:** intercalation, external binding, transport, inner filter effect, micelles, liposomes, hydrophobicity, enthalpy-entropy compensation

## Abstract

The study of the interaction of persistent organic pollutants with biosubstrates helps to unravel the pathways for toxicity, however, few mechanistic data are present in the literature for these systems. We analyzed the binding of paraquat (PQ) and diquat (DQ) herbicides to natural calf thymus DNA and a DNA G-quadruplex by spectrophotometric titrations, ethidium bromide exchange tests, viscometry, and melting experiments. The interaction with bovine serum albumin (BSA) protein was studied spectrofluorimetrically at different temperatures. The retention of the targets on positive, negative, and neutral micellar aggregates and liposomes was analyzed by ultrafiltration experiments. Despite some favorable features, PQ and DQ only externally bind natural DNA and do not interact with DNA oligonucleotides. Both herbicides bind bovine serum albumin (BSA). PQ binds BSA mainly according to an electrostatics-driven process. However, ultrafiltration data also show that some hydrophobic contribution participates in the features of these systems. The practical problems related to unfavorable spectroscopic signals and inner filter effects are also discussed. Overall, both herbicides show a low affinity for nucleic acids and weak penetration into liposomes; in addition, the equilibrium constants values found for BSA system suggest optimal conditions for transport in the body.

## 1. Introduction

**Figure 1:**
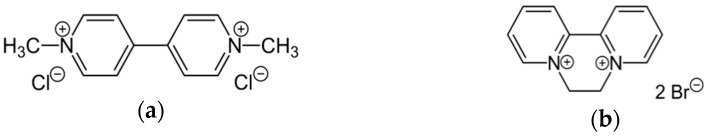
Molecular structures of (**a**) paraquat (PQ) and (**b**) diquat (DQ) herbicides [1].

Among the copious classes of pesticides, bipyridyl herbicides deserve high interest due to their widespread application: they are quaternary ammonium compounds (also known as “quats”) marketed as contact herbicides and desiccants [1]. The paraquat (PQ) and diquat (DQ) molecules that are the focus of the current study (Figure 1) are major components of this family, and both show significant toxicity [2,3,4].

Quat herbicides have high soil adsorption coefficients, suggesting the presence of significant immobilization in this media [5]. In this sense, bioavailability and long-term exposure are not likely to occur. Nonetheless, the retention of cationic herbicides may be modulated by a strongly competitive ion such as copper and other factors which may non-negligibly affect adsorption in soils [5] and the transfer of pesticides to food (as oils) is a matter of study [6]. PQ residues in food are usually not easily detectable and some foods have been reported to have levels of up to 0.2 mg/kg, whereas the acceptable daily intake is 0.004 mg/kg [7]. In addition, different papers evidence human illnesses associated with PQ/DQ acute exposure and poisoning [8,9,10,11]. Other studies enlighten the effect of an exposure associated with multiple pesticide application events resulting in multiple short-term or pulse exposures. For instance, data on early-life stage rainbow trout demonstrated the acute toxicity of DQ on aquatic organisms and enlightened a complex toxicity pathway which significantly changes protein cellular levels, involving proteins related to many different themes, including DNA/RNA polynucleotide processes [12]. In addition to the research connected to the concerns for the detrimental effects of PQ and DQ, it may be cited that they also find use as reference toxic species to test the pharmaceutical effects of plant extracts and medicines [13]. For instance, PQ-induced neurotoxicity is lowered in the presence of extracts of *Bougainvillea glabra* leaves which exert a significant in vivo neuroprotective activity [14], whereas the broad-spectrum anti-fibrotic drug pirfenidone was tested to reduce liver damage by PQ poisoning [15]; furthermore, the polyphenol resveratrol attenuated intestinal damage induced by oxidative stress in DQ-challenged piglets [16]. On that basis, the detailed analysis of PQ and DQ interaction with biosubstrates deserves interest to better understand their effects and their toxicity [17].

PQ was found to be not only toxic but genotoxic [18,19]. It can be bioaccumulated, for instance in kidneys [20] and lungs [21], and its bioaccumulation/chronic use is also supposed to be related to the onset of Parkinson’s disease [7,14]. PQ toxic effects are principally due to redox reactions that convert the herbicide into active free radicals [22]. The fast oxidation of these species leads the cells to death, through the formation of superoxide ions O^2−^ whose detrimental action is widely discussed [23]. PQ-induced oxidative stress was found to induce DNA methylation variations through reactive oxygen species (ROS) production [24]. DQ herbicidal action on plant cells is also due primarily to the initiation of ROS formation, lipoperoxidation, and apoptotic cell death [25,26]. DQ may have teratogenic effects [27] and prolonged exposure to DQ affects kidneys, brain, and the gastrointestinal tract [28].

The damage generated by free radicals/ROS affects DNA molecules, proteins, and membrane phospholipids [7]: a careful check if PQ and DQ quats may interact with these biosubstrates, not only via ROS but by some direct effect, deserves interest. Note that binding to polynucleotides is also interesting from the point of view of herbicide detection: addressing the need for accurate quantification of persistent pollutants in environmental samples, sensitive biosensors constructed using DNA molecules exhibited an ultrasensitive response to DQ [29,30]. In addition, the interaction with golden standard reference proteins as the abundant serum albumins needs to be tested because the correlation between albumin’s binding and biodistribution has been highlighted by several studies [31]. Methyl parathion/albumin interaction has crucial importance in the pesticide’s toxic activity [32] and the reversible binding to bovine serum albumin can be exploited to enhance a pesticide’s performance [33]. To the best of our knowledge, only an example of a PQ/DNA binding study is reported in the literature [34] where PQ is proposed to weakly bind the DNA groove. However, the authors do not seem to consider the superimposition of the spectroscopic signals, which strongly affect and limit the investigation. No mechanistic study on DQ/DNA interaction is available, and information on cytotoxicity comes from biological analyses only [35,36]. PQ and DQ have been tested for affinity to human serum albumin (HSA) [37,38]. Notwithstanding this previous research, the crucial importance of the inner filter effects and the bias produced by their presence does not always emerge from these few studies and some binding details, in addition to the preferential binding site, have not been discussed. Finally, regarding the point of view of the lipid target, there is a need to investigate the interaction of polar pesticides with cell membranes to help to determine their toxicity mechanism. Recent studies showed the interest in testing the affinity to liposomes because these data may also complete information on bioaccumulation, which should not only be based on the hydrophobic nature of the compounds (partition octanol-water) [39]. Indeed, biophysical studies on the lipid model system which mimics some of the features of natural membranes and on membrane interactions by exogenous species provide important information on the possible mode of action of both healthy and toxic species [40]. For instance, antimicrobial peptides are found to strongly alter the characteristics of lipid bilayers: these interactions are crucial for the activity of the antibiotics and studies aimed at enlightening the details of these processes are crucial for developing improved pharmaceuticals [41,42]. 

Overall, despite the long-lasting high interest in the persistent interaction of organic pollutants with biosubstrates, a detailed chemical analysis of the mechanism of binding to possible direct biotargets, such as polynucleotides, proteins, and lipid membranes, is still uncomplete. Within the context of the national project of research in Antarctica (PNRA), the current study followed previous work on pesticides [43] and was devoted to investigating the interaction between PQ and DQ herbicides and biosubstrates to provide mechanistic details on their possible toxic pathways. The spectroscopic experimental setup was carefully designed to ensure the reliability of the results under unfavorable conditions, a crucial aspect that is sometimes underestimated. Moreover, the retention percentage on micelles and liposomes was evaluated for PQ and DQ; these tests constitute a first basis for the estimation of the affinity for the cellular membrane. 

Therefore, the aim of the experiments reported here was both to discuss a robust procedure to analyze the details of the binding mechanism and provide further insight into whether, parallel to ROS production, the toxicity of these species may be connected to some direct binding or affinity for polynucleotide, oligonucleotide, protein, or membrane interactions. 

## 2. Materials and Methods

### 2.1. Materials

Paraquat dichloride hydrate (PQ, purity ≥ 98%) and diquat dibromide monohydrate (DQ, purity ≥ 95%) were supplied by Sigma. Stock solutions (ca. 1 mM) were obtained by dissolving known amounts of the solid in water. Their molar concentration will be indicated as C_D_ (dye).

The DNA used is that extracted from calf thymus (from now on ct-DNA) and consists of 41.9 mol% G–C and 58.1 mol% A–T base pairs. Lyophilized sodium salt from Sigma was dissolved in water and sonicated (MSE-Sonyprep sonicator, 7 cycles of 10 s sonication +20 s pause at an amplitude of 14 µm, solution kept in an ice bath) [44]. Gel electrophoresis was used to determine the length of the fragments, being approximately 500 base pairs (100 bp DNA ladder is used as the reference). Stock solutions of ct-DNA were standardized spectrophotometrically (ε = 13,200 M^−1^ cm^−1^ at λ = 260 nm, I = 0.10 M, pH = 7.0—for the molar extinction coefficients see [45] and references therein); concentrations of ct-DNA are expressed in molarity of base pairs as C_P_ (polynucleotide). The dried DNA oligonucleotide 5′-TAGGGTTAGGGTTAGGGTTAGGG-3′ (Tel23—hybrid, telomeric) was purchased from Metabion; its stock solution was prepared in aqueous buffer, and the molar concentration (in strands) was calculated according to the weight/content provided by the sample certificates. The formation of the G-quadruplex (G4) structure was carried out by heating oligonucleotide solutions to 90 °C for 6 min and slowly cooling to room temperature. Bovine serum albumin (BSA) was provided by Sigma as a crystallized and lyophilized powder (≥98%, agarose gel electrophoresis and ≤0.005% fatty acids). Known amounts of lyophilized solid were dissolved in water, and the molar concentration of the protein (ca. 0.1 mM) was determined by measuring light absorption using absorptivity (ε = 44,000 M^−1^ cm^−1^ at λ = 278 nm), also herein indicated as C_P_ (protein).

Ethidium bromide (EB), solid (purity > 99%) was from Sigma; its stock solutions were prepared by weight but the concentration was spectrophotometrically checked (ε = 5700 M^−1^ cm^−1^ at λ = 480 nm, I = 0.10 M, pH = 7.0). Surfactant solutions in concentrations higher than their critical micellar concentrations (CMC) were used to ensure the presence of the micellar structures. Stock solutions of sodium dodecylsulphate (SDS) (0.2 M, Sigma Aldrich, St. Louis, MI, USA, CMC = 8 × 10^−3^ M), dodecyltrimethylammonium chloride (DTAC) (0.2 M, Sigma Aldrich, CMC = 1 × 10^−2^ M) and Triton X-100 (0.02 M, Sigma Aldrich, CMC = 2 × 10^−4^ M) [46] in ultra-pure H_2_O were suitably diluted to obtain the working solutions. The monomer 2-oleoyl-1-palmitoyl-sn-glycero-3-phosphocholine (POPC, Sigma Aldrich, purity 95.5%) was used for the formation of the liposomes. A stock solution of about 5 × 10^−4^ M was prepared by the procedure described below, and working solutions were obtained from its dilution. About 5 mg POPC was solubilized in 10 mL of absolute MeOH, obtaining a clear solution. The solution was then subjected to drying under nitrogen flow until the complete evaporation of the organic solvent. The vial was further connected to a water pump to ensure the removal of any solvent residues. A thin film was obtained: the addition of an aqueous medium (10 mL of aqueous buffer plus stirring for a few minutes) caused the swelling of the film and the consequent detachment from the surface of the vial. The spontaneous aggregation of POPC in the aqueous phase leads to the formation of multi-lamellar vesicles (Large Multi-lamellar Vesicle, LMV), in which concentric phospholipid bilayers are separated by water. The solutions were stored in a refrigerator at 4 °C.

All the tests were carried out in aqueous solution buffered with sodium dimethylarseniate (NaCac, sodium cacodylate) 2.5 mM for pH 7.0. The used buffer also contained NaCl, which was used as the salt medium (exception for DNA melting); for the G-quadruplex LiCac and KCl were used in place of NaCl and NaCac respectively (LiCac was obtained by mixing suitable amounts of LiOH and HCac, both from Sigma). All of the solutions used were made in ultra-pure grade water, obtained from distilled water further subjected to deionization and sterilization using the AriumPro system (Sartorius).

### 2.2. Methods

**Figure 2:**
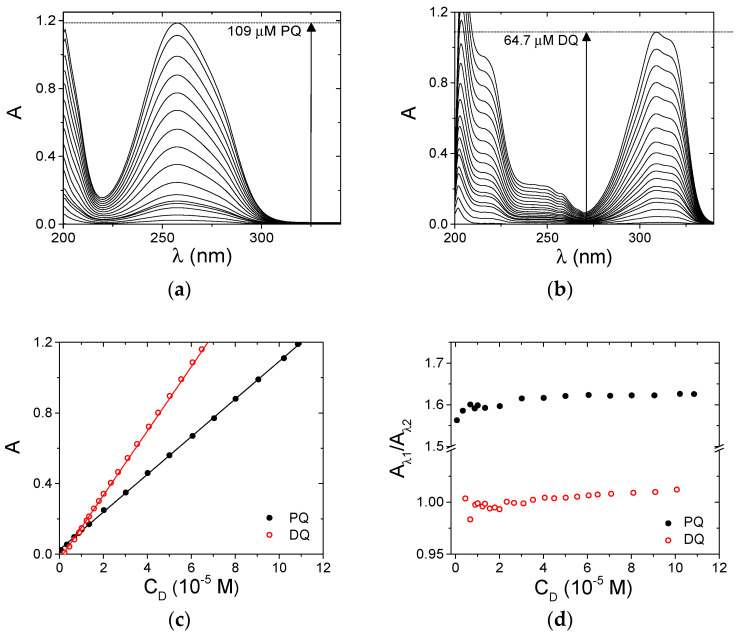
Spectroscopic characterization of PQ and DQ, NaCl 0.1 M, NaCac 2.5 mM, pH 7.0, 25.0 °C. (**a**) PQ from 4.08 × 10^−7^ to 1.09 × 10^−4^ M; (**b**) DQ from 2.26 × 10^−7^ to 6.47 × 10^−5^ M; (**c**) relevant Lambert–Beer plots (full mark is PQ at 258 nm ε = 1.07 × 10^4^ M^−1^ cm^−1^; open mark is DQ at 309 nm ε = 1.82 × 10^4^ M^−1^ cm^−1^; (**d**) absorbance ratio plots (PQ full mark, A258 nm/A280 nm; DQ open mark, A297 nm/A324 nm.

A Shimadzu UV-2450 (Shimadzu Corporation, Kyoto, Japan) double ray spectrophotometer was used to record absorption spectra and to perform spectrophotometric titrations. The fluorescence experiments were carried out by employing a Perkin Elmer LS55 spectrofluorometer (Perkin Elmer, Waltham, MA, USA) [47]. The excitation light is provided by a pulsed Xenon lamp (50 Hz). The instruments are equipped with temperature control within ± 0.1 °C. The measurements were performed by employing quartz cells of minimum content needed equal to 500 or 1000 μL, with an optical path length of 1.0 cm. In the spectrophotometric titrations, increasing amounts of the titrant were added directly in the cuvette containing the titrand and a spectrum was recorded upon each addition. The precise and accurate addition of very small volumes was done using a glass syringe connected to a Mitutoyo (Mitutoyo Italy, Milan, Italy) micrometric screw (one complete turn of the screw is 8.2 µL, 1/50 of a turn is the minimum addition possible). In the case of fluorescence, inner-filter effects were corrected according to the procedure detailed in the text (see Equation (2) below); for these corrections we used the values of molar extinction coefficient ε_PQ_ = 1548 M^−1^ cm^−1^ at λ_ex_ = 295 nm and the ε_PQ_ at each of the emission wavelengths extracted by the experiment shown in Figure 2a.

The viscosity of the solutions was measured with a Ubbelohde viscometer (Merck, Darmstadt, Germany) immersed in a controlled temperature bath. The temperature was kept constant at T = 25 ± 0.1 °C. A quantity of 3.0 mL of ct-DNA solution of approximately 2.0 × 10^−4^ M was used. Flow times of ligand/DNA mixtures at different C_D_/C_P_ ratios were recorded by adding increasing volumes of ligand directly to the polynucleotide solution. The systems were carefully mixed by sucking and blowing the liquid inside the capillary. Control experiments were performed by adding the same volumes of buffer solution to take into account possible dilution contributions on the DNA viscosity. The flow time of the solvent was also measured. All of the measurements were repeated at least 5 times. The relative viscosity of the polynucleotide was calculated as follows (Equation (1)):

(1) $$\frac{\eta_{\mathrm{sample}}}{\eta_{\mathrm{reference}}}=\frac{\eta }{\eta_{0}}=\frac{t_{\mathrm{sample}}{-t}_{\mathrm{solvent}}}{t_{\mathrm{reference}}{-t}_{\mathrm{solvent}}}$$

where t_sample_, t_reference_, and t_solvent_ are respectively the observed flow times for the herbicide/DNA mixtures, DNA alone, and the buffer solution. The relative viscosity is connected to the polynucleotide elongation as L/L_0_ = (η/η_0_)^1/3^, where L is the length of the bound polynucleotide and L_0_ is the length of the free one.

The melting experiments were undertaken spectrophotometrically on the Shimadzu UV-2450 (Shimadzu Corporation, Kyoto, Japan) double ray apparatus, by heating the sample from 25 °C to 90 °C with a scan rate of +5 °C/min every 6.5 min. After keeping the temperature constant for 6.5 min to allow the system to reach the equilibrium, an absorbance spectrum was recorded. Thermal denaturation curves were obtained by monitoring absorbance changes at λ = 260 nm for DNA and 295 nm for Tel23 G-quadruplex. They are plotted as the percentage of the absorbance change%ΔA = 100 × (A − A_in_)/(A_fin_ − A_in_), where A_in_ and A_fin_ represent the absorbance values at the initial (low temperature) and final (high temperature) plateau. The melting temperature (T_m_) was extrapolated as the inflexion point of the resulting sigmoidal trend, and the difference between the T_m_ of ligand + polynucleotide and the T_m_ of the polynucleotide represents the ΔT_m_ value.

In this work, the fraction of pollutants retained on the surfaces of micellar structures (SDS, DTAC, TritonX) and liposomes (POPC) was evaluated by ultrafiltration experiments undertaken using an Amicon 8050 (Merck Millipore, Darmstadt, Germany) stirred ultrafiltration cell (capacity 50 mL). The analyzed mixture was forced to pass through an Amicon cellulose membrane (9 mL over initial 10 mL inserted in the cell; membrane diameter 4.45 cm, surface 13.4 cm^2^) with a 3000 Dalton cut-off at a constant pressure of 40 psi and 300 rpm. Comparison between the spectrophotometrically quantified pollutant content before (A_in_) and after (A_fin_) filtration (at the wavelength which corresponds to the maximum absorbance of the dye) enables the evaluation of the percentage of retention as R% = 100 × (A_in_ − A_fin_)/A_in_). All measurements were performed in triplicate.

The density functional theory (DFT) calculations were performed using the Gaussian 16 package [48]. The Integral Equation Formalism (IEF) version [49] of the Polarizable Continuum Model (PCM) [50] was used to describe the implicit effects of the solvent (water). The ground state geometry optimizations were performed using the B3LYP functional and the 6-31G(d) basis set. For the docking calculations, the binding sites were constructed on the actual position of the selected markers. For binding site I, PDB id 2BXC was employed as starting structure, whereas PDB id 2BXG was used for binding site II. Spheres of radius 0.14–0.4 nm were created in the place of the ligands in a 0.7 nm large box. The grid-based score depends on the non-bonded terms of the molecular mechanics force field. The ligand charge for the docking was calculated using the AM1-BCC method.

## 3. Results and Discussion

### 3.1. Spectroscopic Characterization

The optical properties of PQ and DQ molecules were characterized under physiological conditions (NaCl 0.1 M, NaCac 2.5 mM, pH 7.0). Absorbance spectra at different concentrations were recorded for both of the herbicides (Figure 2a,b). The unaltered spectral profile (Figure 2a,b) and the linearity of the Lambert and Beer plots (Figure 2c) indicate that the molecules do not aggregate under the explored conditions. The possible presence of aggregates was checked by inspecting absorbance ratio plots which may evidence a more subtle change in the profiles (Figure 2d). 

The constancy of the plotted values further confirms the absence of self-aggregation processes. The lower aggregation tendency shown here with respect to the previously analyzed pollutants [43] agrees with the +2 charge borne by PQ and DQ.

### 3.2. ct-DNA Binding

**Figure 3:**
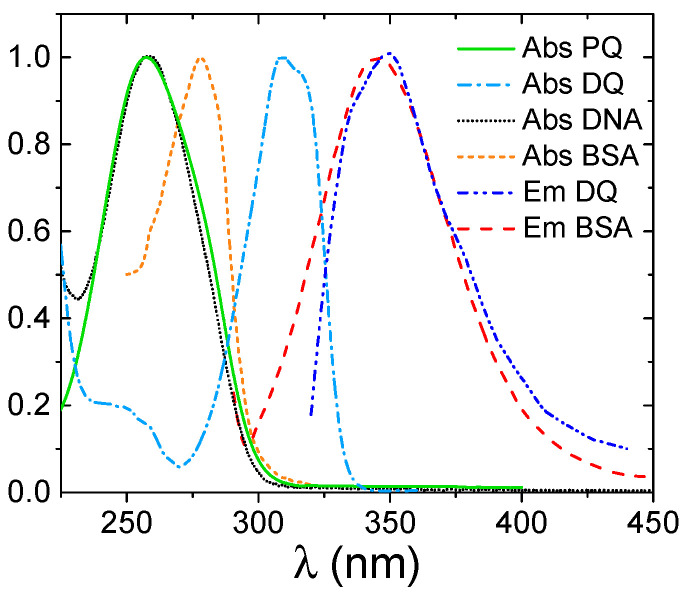
Normalized absorbance (Abs) and emission (Em) profiles of PQ, DQ and the biotargets.

In principle, the non-aggregation of the dye is a highly favorable aspect for the further analysis of its interaction with the target. Nonetheless, the analysis of the binding of PQ and DQ to nucleic acids and proteins unfortunately still remained highly complex. The main obstacle to the spectroscopic investigation was the superimposition of the pollutants’ signals with those of the biosubstrates (Figure 3).

Under these circumstances, in the case of absorbance experiments, differential titrations may be performed: the same amount of the titrant (DNA) is added to both the measuring and the reference cell. Figure S1 (Supplementary Materials) shows an example of the spectra recorded for differential titrations. As far as the DNA content is raised and therefore high values of DNA absorbance are reached, this approach may be biased and thus not highly reliable. However, neither PQ nor DQ showed significant absorbance profile changes or bathochromic effects, even for the first steps of DNA additions. This finding may be the first hint of a scarce interaction between the herbicides and DNA.

To overcome the problem, fluorescence exchange titrations with EtBr were performed where known amounts of herbicides were added directly to EtBr-saturated DNA. A decrease in emitted fluorescence at the excitation and emission wavelengths typical of intercalated EtBr would have indicated its displacement from the helix [51]. Although the selected herbicides present suitable features to be considered as DNA binders, the tests do not demonstrate any significant interaction (example in Figure S2), meaning that intercalation is excluded. Groove binding also seems unlikely, because the penetration within the groove (and even more of a charged species) is usually still able to produce some change in the EtBr probe environment which is reflected by a signal change [52].

**Figure 4:**
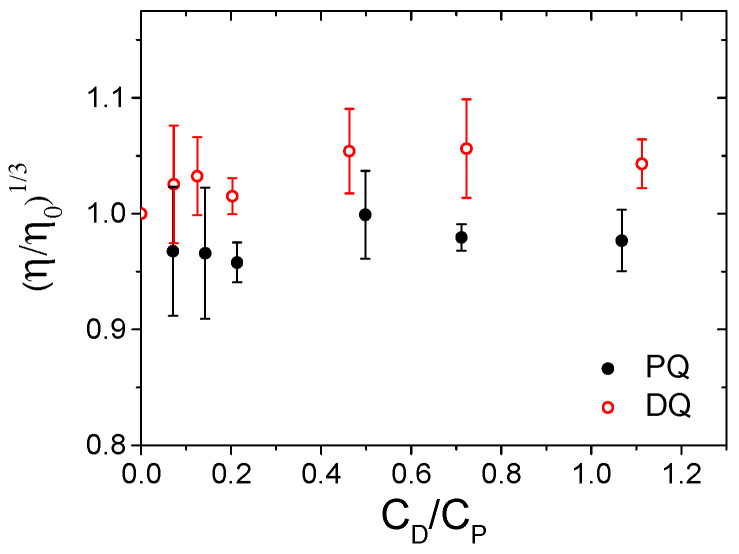
Relative viscosity vs. herbicide/DNA ratio (C_D_/C_P_) for PQ (full mark) and DQ (open mark); C_DNA_ = 1.77 × 10^−4^ M, C_PQ_ from 0 to 1.92 × 10^−4^ M, C_DQ_ from 0 to 2.0 × 10^−4^ M, NaCl 0.1 M, NaCac 2.5 mM, pH 7.0, 25.0 °C.

The same conclusion emerges from the viscosity measurements carried out for the two herbicide/DNA systems at different herbicide/DNA (C_D_/C_P_) ratios. The flow times (t) were used to calculate the relative viscosity according to Equation (1). For both herbicides, the relative viscosity remained almost constant upon the addition of increasing amounts of herbicide (Figure 4), indicating no significant elongation of the DNA helix. This result underlines the absence of intercalation between the DNA base pairs for both PQ and DQ.

**Figure 5:**
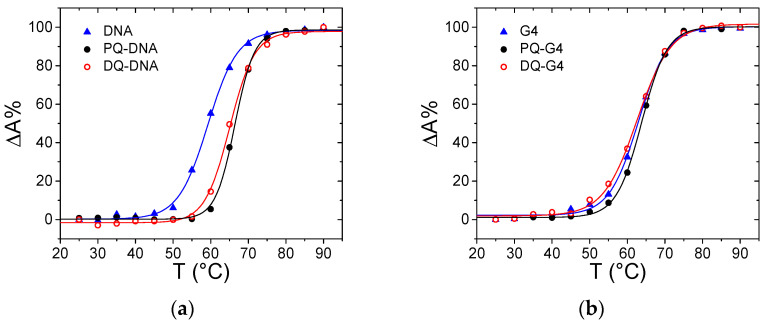
Melting curves for (**a**) ct-DNA (triangle), PQ/DNA (full circle) and DQ/DNA (open circle), C_DNA_ = 1.14 × 10^−5^ M, C_PQ_ = 1.14 × 10^−5^ M, C_DQ_ = 1.06 × 10^−5^ M, NaCac 2.5 mM, pH 7.0, 260 nm; (**b**) G-quadruplex tel23 (G4, triangle), PQ/G4 (full circle) and DQG4 (open circle), C_G4_ = 5.51 × 10^−6^ M, C_PQ_ = 6.01 × 10^−6^ M, C_DQ_ = 5.47 × 10^−6^ M, KCl 0.1 M, LiCac 2.5 mM, pH 7.0, 295 nm.

Thermal denaturation studies revealed that the stability of DNA is affected by the presence of the ligands (Figure 5a): a stabilizing effect on the melting temperature of the polynucleotide is observed upon the addition of the herbicides (ΔT_m_ ca. +6 °C for both PQ and DQ). This result indicates that some non-negligible interaction is involved. However, based on the previous results, an external interaction between the +2 charged PQ and DQ and the negatively charged external helix backbone may be considered as the most probable option. Note that this, even weak, interaction is found to be present for double-stranded DNA only. Absorbance titrations performed on a G-quadruplex forming oligonucleotide gave negative results for binding (no signal change, Figure S3). In addition, melting tests do not evidence any stabilizing effect (Figure 5b).

**Figure 6:**
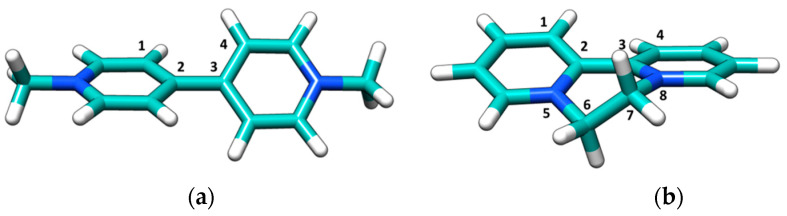
Density functional theory (DFT) optimized molecular structure of (**a**) PQ and (**b**) DQ.

These systems could, in principle, fit those of common DNA binders: the small, aromatic geometry and the presence of +2 positive charges suggests possible intercalation into the DNA pocket. To refine the results which indicate the non-intercalation between DNA base pairs, DFT calculations were performed on both PQ and DQ to obtain their geometrical structure (Figure 6). The two aromatic rings are tilted (Table 1), producing some steric hindrance: an energy penalty would be paid to reach the planarization needed for a better accommodation in the helix core (for natural DNA base pairs planes are at a 3.4 Å distance [53], each of the bonds of Figure 6 lies in the 1.4–1.5 Å range). This might contribute to the low affinities observed; note that a series of DQ derivatives with substituents which widen the species’ planarity were found to switch their binding mode from electrostatic to intercalation [17]. 

### 3.3. BSA Binding

#### 3.3.1. Spectrofluorometric Titrations

For testing of the binding of the herbicides to proteins, absorbance approaches have to be avoided due to the already discussed experimental issues. Concerning fluorescence techniques, the superimposition of the emission signals of DQ and BSA significantly complicates the spectroscopic analysis of the DQ/BSA system and prevents it from being performed with high accuracy.

**Figure 7:**
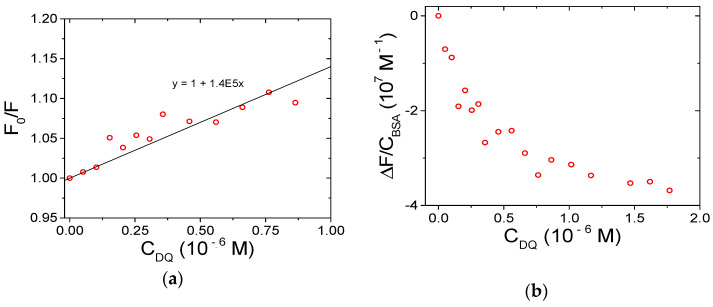
Spectrofluorimetric titration for the DQ/BSA system: (**a**) Stern–Volmer plot; (**b**) binding isotherm; C_BSA_ = 1.50 × 10^−6^ M, NaCl 0.1 M, NaCac 2.5 mM, pH 7.0, 37.0 °C, λ_ex_ = 295 nm, λ_em_ = 345 nm.

Correction for the emission intensity of DQ is not straightforward because the corresponding equation (see Equation (2) below) holds only for not-too-distorted signals (A < 0.05–0.1; [54,55]). The bias is enhanced by the fact that DQ emission overlaps the spectral range where BSA quenching is followed. Therefore, during a titration where DQ is added to BSA and for DQ excess (which will hold even more as the titration proceeds), the fluorescence read will be the sum of the emission free BSA, free DQ, and DQ/BSA adduct (Figure S4). Any attempt to deconvolute the different contributions produces highly biased results. However, data robustly demonstrate that the binding does indeed take place (Figure 7). To limit the problems cited above, the first points only of the titration may be used and analyzed (with HypSpec2014^®^ software (Hyperquad, Leeds, UK). See below PQ for additional details, data treatment for DQ is provided in Figure S5. This procedure yields a binding constant of the magnitude order of 10^6^ M^−1^ for the DQ/BSA system.

**Figure 8:**
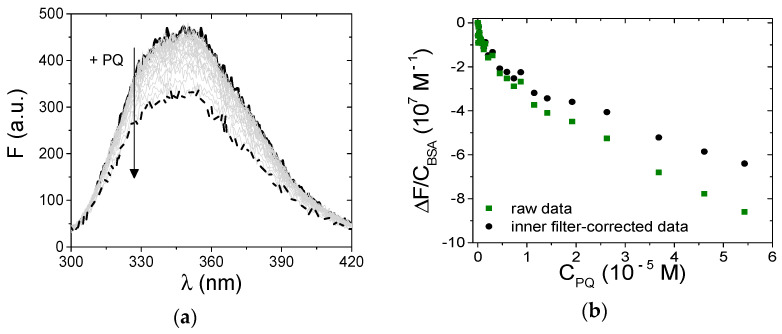
(**a**) Spectrophotometric titration of PQ/BSA and (**b**) corresponding binding isotherm at λ_em_ = 345 nm; C_BSA_ = 1.50 × 10^−6^ M, C_PQ_ from 0 (solid) to 5.43 × 10^−5^ M (dash), NaCl 0.1 M, NaCac 2.5 mM, pH 7.0, 25.0 °C, λ_exc_ = 295 nm; squares refer to uncorrected fluorescence values, circles define the trend corrected according to the equation in the text.

Differently, in the case of PQ, the spectrofluorometric titrations could be carried out under more favorable conditions that enabled analysis of the details of the interaction with BSA. Note that inner filter effects still affected the measurements: we carefully optimized the experiments and corrected the signals according to:

F_corr_ = F_obs_ × 10^(Aex+Aem)/2^ (2)

where F_corr_ and F_obs_ are, respectively, the corrected and the observed fluorescence intensities, and A_ex_ and A_em_ are the absorbance values, respectively, at the excitation and emission wavelengths [54]. In particular, A_ex_ is the optical density of PQ at the excitation wavelength (295 nm) and A_em_ is its value at λ_em_, which will change point by point of the spectrum to yield the corrected one. In principle, the total absorbance of the mixture should be considered. However, because BSA absorbance is negligible in the spectral range studied and PQ is present in excess (bound PQ minority), the correction can be done using the molar extinction coefficients of free PQ in the buffer (Figure 2a). Figure 8a shows the BSA emission spectral changes observed upon the addition of increasing amounts of PQ; Figure 8b indicates evident deviations in the recorded binding isotherm (λ_em_ = 345 nm) in comparison to that corrected for the inner filter effect. Taking into account this evidence, the intensities of the BSA fluorescence were corrected for inner filter effects before any further data analysis.

**Figure 9:**
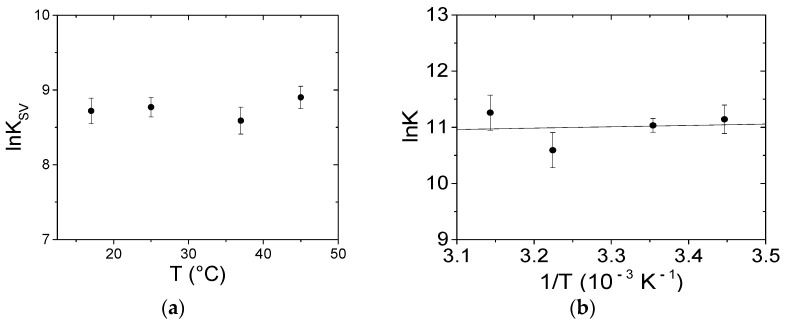
Dependence on temperature of (**a**) the Stern–Volmer constant K_SV_ and (**b**) the binding constant K for the PQ/BSA system; NaCl 0.1 M, NaCac 2.5 mM, pH 7.0.

To ensure that the fluorescence decrease was not due to collisional quenching only, data recorded at different temperatures were fitted using the Stern–Volmer equation (Equation (3)):

(3) $$\frac{F_{0}}{F}=1+k_{q}\tau_{0}=1+K_{\mathrm{SV}}\left[Q\right]$$

where F_0_/F corresponds to the ratio between the BSA fluorescence intensity in the absence and the presence of the quencher (PQ), respectively, K_SV_ represents the Stern–Volmer constant, [Q] is the molar concentration of the quencher, k_q_ is the bimolecular quenching constant, and τ_0_ corresponds to the average lifetime of the protein in the absence of quencher. Note that the F values must be corrected for dilution and inner filter before use. For static quenching, K_SV_ will be equal to the binding constant for complex formation (if the complex is non-fluorescent). Note that [Q] corresponds to [Q_free_] (the molar concentration of free quencher). Therefore, just the points under quencher excess ([Q_free_] ≅ Q_tot_) were considered in the data analysis. K_SV_ is equal to (6.5 ± 0.8) × 10^3^ M^−1^ at 25.0 °C. Because τ_0_ = 7 ns for protein BSA [56], k_q_ results are beyond the upper limit for collisional quenching (k_q_ = 3 × 10^10^ M^−1^ s^−1^) and should be necessarily related to the presence of some non-collisional quenching (complex formation) [55]. Moreover, the lack of dependence on temperature (Figure 9a) also confirms the non-collisional nature of the quenching process.

The binding constants at the different temperatures were calculated using HypSpec2014^®^ software, which enables, through a least squares procedure, fitting the data over a wavelength range according to multiple equilibria models (Figure S6). Note that all of the spectra were previously corrected for the inner filter effect over the whole explored range at each of the A_λex_ and A_λem_ appropriate for each point of each spectrum. Tests for different models and factor analysis of the data suggest that a 1:1 binding is sufficient to describe the data set. At 25.0 °C a binding constant (K) of (6.2 ± 0.8) × 10^4^ M^−1^ was measured. Figure 9b shows that the obtained K values do not significantly change with temperature, indicating that the enthalpy variation (ΔH) is negligible; on the other hand, the entropy variation (ΔS) was equal to 85 J/mol·K. Therefore, the driving force for binding is due to the entropic term with a negligible enthalpy contribution to the process. The sign and the order of magnitude of the thermodynamic parameters constitute a signature for the binding type [55]: in our case the binding is supposed to be mainly driven by electrostatic forces, even if some contribution of a hydrophobic interaction cannot be completely ruled out (see retention tests below). Interestingly, the thermodynamic parameters extracted from the literature lie on a common line [55]. This correlation is called enthalpy–entropy compensation (EEC). EEC is a phenomenon which has also been attributed to experimental bias or intended as a simple result of thermodynamic laws [57]. Currently, most researchers agree on EEC which is connected to the fact that, if a small molecule undergoes more and/or tighter van der Waals contacts and H-bonds with the substrate (a process related to a more negative ∆H), this will produce a decrease in the flexibility in one or both ligand-substrates. Overall, the reduction in the overall conformational entropy will compensate for the enthalpy decrease [58]. Note that hydration also plays a major role: the rearrangement in the coordinated solvent molecules strongly influences, in particular, ΔS [58,59]. The correlation plot of Figure S7 for ligand-BSA systems yields a linear relationship with a slope close to one. This means that the enthalpy gain is compensated for the entropic loss. This compensation is always found in the case of flexible macromolecules, but significantly lower slopes can be found for stiff hosts [60]. The thermodynamic values obtained for the PQ/BSA system agree with an electrostatics-driven process, in agreement with the charged nature of the host.

#### 3.3.2. BSA Binding Site

**Figure 10:**
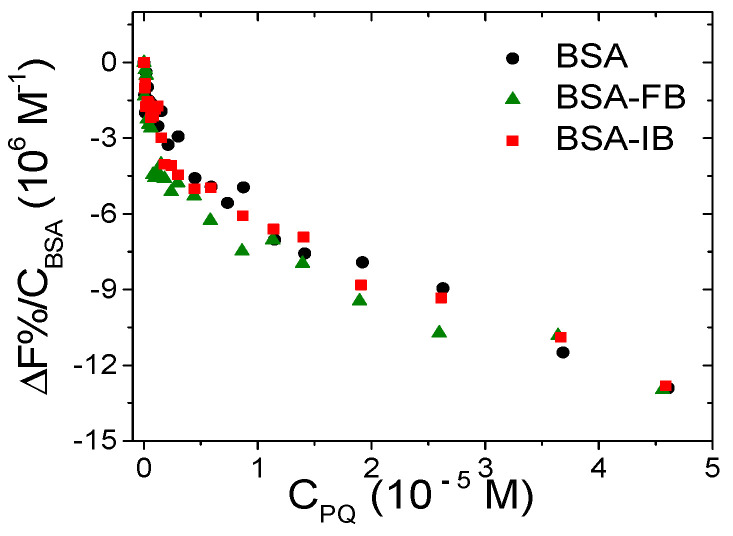
Binding isotherm for free bovine serum albumin (BSA) and BSA/marker titrated with PQ (FB = phenylbutazone, IB = ibuprofen); C_BSA_ = 1.5 × 10^−6^ M, C_marker_ = 1.5 × 10^−5^ M, C_PQ_ from 0 to 4.6 × 10^−5^ M, NaCl 0.1 M, NaCac 2.5 mM, pH 7.0, 25.0 °C, λ_exc_ = 295 nm, λ_em_ = 345 nm.

BSA possesses two main binding sites [61] and the evaluation of the preferential binding position is usually obtained through fluorescence competitive studies. Phenylbutazone (PB) and ibuprofen (IB) are species employed, respectively, as site I and II markers, and were chosen for the current study. Figure 10 shows the binding isotherms for PQ obtained by titrating BSA alone, BSA saturated with PB, and BSA saturated with IB. The negligible difference observed in the trends suggests that the binding is not selective.

**Figure 11:**
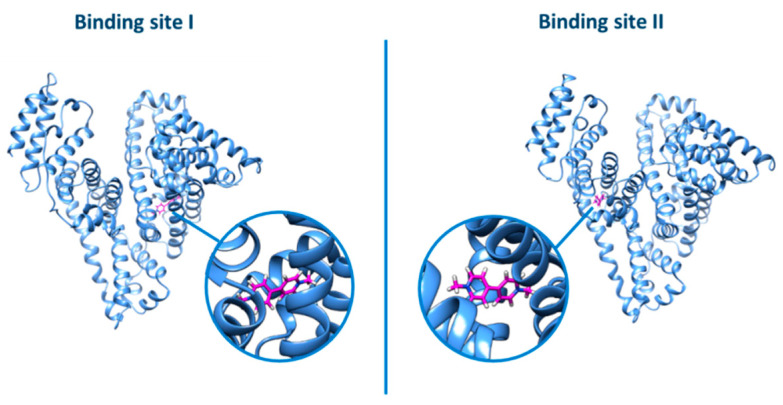
Docking of PQ in binding sites I or II of BSA.

The same picture was also evidenced by the docking analysis (Figure 11): no relevant difference was observed by docking the ligand into the two different binding sites (grid score for binding site I = −26.16; grid score for binding site II = −26.46).

Overall, it appears that PQ, which is a small charged species, is bound by BSA by an electrostatic, non-specific process as for the preference between site I or site II. 

### 3.4. Micellar Enhanced Ultra-Filtration (MEUF) Tests on Surfactants and Liposomes

The retention of PQ and DQ on micelles of different nature (positive, negative, or neutral surface) and liposomes was studied as an indication of lipophilicity and affinity for cellular membranes. Sodium dodecyl sulphate (SDS) and dodecyl trimethyl ammonium chloride (DTAC) were used respectively for the positively and negatively charged micelles. TritonX-100 was employed for the neutral micelles, whereas 1-palmitoyl-2-oleoyl-sn-glycero-3-phosphocholine (POPC) molecules composed the liposomes. Micellar Enhanced Ultra-Filtration (MEUF) coupled with absorbance spectroscopy enables the percentage of retention (R%) on the micelles/liposomes to be measured (see Section 2). Figure S8 shows an example of the absorbance spectra of PQ/SDS and DQ/DTAC solutions recorded before and after the ultrafiltration process. The analysis was performed at two different ionic strengths (NaCl 0.1 or 0.5 M, NaCac 2.5 mM, pH 7.0) and the obtained results are reported in Table 2.

The positively charged herbicides are strongly retained on the negative surface of the SDS micelles but the increase in the salt content strongly affects the electrostatic nature of the binding, resulting in a significant decrease in R%. Based on electrostatics, PQ and DQ should not interact with the positive surface of DTAC. On the contrary, even with low R%, PQ and DQ are both still retained on DTAC micelles and the retention is scarcely affected by the variation of the ionic strength. This evidence suggests the presence of some hydrophobic forces also involved in the binding which, even if minor (+2 charged species), would be related to the aromatic rings present in the molecular structure. 

The same hint is provided by the R% values obtained for the neutral TritonX micelles. The low hydrophobicity of the systems, octanol-water partition coefficient logP_ow_ = −4.22 for PQ [62] and logP_ow_ = −4.6 for DQ, [63] prevents any strong affinity, but interaction is observed nonetheless. Regardless of the salt content, the adsorption on POPC liposomes results are similar to those obtained for DTAC. It may be speculated that the common terminal –N–(CH_3_)_3_^+^ residue in DTAC and POPC plays an important role in driving these similarities. However, ultrafiltration experiments alone are not sufficient to robustly extract this information and are only preliminary tests. Future development of these aspects should consider studies using different liposomes (differently charged, zwitterionic, or even lipid mixtures and supported lipid bilayers). Such detailed studies on lipid model systems mimicking the cellular membrane would allow various information to be obtained, such as the affinity towards regions with different degrees of hydrophobicity, the role played by the different charges, and the binding-induced changes of the membrane mechanical properties [40,41,42].

## 4. Conclusions

In this study, we tried to provide evidence that, for systems with no significant visible absorption, a careful evaluation of the superimposition with biosubstrate signals is needed to yield a robust description of the mechanistic aspects for binding. This also holds for the bias caused by inner-filter effects in fluorescence measurements. This will, unfortunately, be true for many molecules of the herbicides/pesticides family. 

Regarding the comprehension of the possible toxic pathways, PQ and DQ appear to behave in a similar way, with no interaction with DNA oligomers and only external interaction with DNA polynucleotides. Therefore, pathways considering direct effects on nucleotides should most likely be rejected. 

By comparison, the capability of herbicides to bind BSA may play a key role in their toxic activity. One of the main functions of serum albumins is their involvement in the transport, distribution, and metabolism of exogenous and endogenous substances [64,65]. The interaction between DQ and PQ with BSA can therefore support the bioavailability, in addition to the spreading of the herbicide in living organisms. Binding constant values in the 10^4^–10^6^ range, as found here, are reported as optimal for the complexation of the ligands and the consequent release once target or appropriate conditions are reached [66].

Data on micelles and, in particular, liposomes, show that, despite the positive charge present in the POPC outer shell, some retention is possible. These preliminary tests open the way to future studies in which the interaction with the membrane is more deeply analyzed by biophysical studies with different types of liposomes.

## Figures and Tables

**Table 1:** Values of some dihedral angles for paraquat (PQ) and diquat (DQ) molecular structure according to density functional theory (DFT) calculations.

	Label	Value (°)
PQ	1-2-3-4	39.4
DQ	1-2-3-4	−22.8
DQ	3-8-7-6	39.9
DQ	2-5-6-7	39.5
DQ	5-6-7-8	−58.3

**Table 2:** Retention percentage (R%) of the analyzed herbicides on micelles and liposomes (NaCac 2.5 mM, pH 7.0, 25.0 °C, C_SDS_ = C_DTAC_ = C_TritonX_ = 0.01 M, C_POPC_ = 5 × 10^−6^ M). Tests were performed in triplicate, errors are ±SD.

	PQ		DQ	
NaCl	0.1 M	0.5 M	0.1 M	0.5 M
SDS	90 ± 1	41 ± 5	91 ± 1	34 ± 5
DTAC	8 ± 1	6 ± 1	3 ± 1	6 ± 1
TritonX	11 ± 2	18 ± 2	15 ± 1	15 ± 1
POPC	5 ± 1	4 ± 2	5 ± 2	6 ± 1

## Data Availability

The raw data presented in this study are not publicly available but are available on request from the corresponding author.

## Supplementary Materials

The following are available online at https://www.mdpi.com/1660-4601/18/5/2412/s1, Figure S1: Spectrophotometric titrations of PQ/DNA and DQ/DNA, Figure S2: Fluorescence exchange titration of EtBr-saturated DNA with DQ, Figure S3: Spectrophotometric titration for the Tel23-DQ system, Figure S4: Spectrofluorimetric DQ/BSA titration, Figures S5 and S6: HypSpec2014 analysis for the titrations of DQ/BSA and PQ/BSA, Figure S7: Plot of ΔH vs. TΔS for different ligands binding to BSA, Figure S8: Absorbance spectra for the ultrafiltration experiments of PQ in SDS and DQ in DTAC.

## References

[1] Eddleston M. (2016). Bipyridyl Herbicides. Critical Care Toxicology.

[2] Lohitnavy M., Chitsakhon A., Jomprasert K., Lohitnavy O., Reisfeld B. (2017). Development of a physiologically based pharmacokinetic model of paraquat. Proc. Annu. Int. Conf. IEEE Eng. Med. Biol. Soc. EMBS.

[3] Moravčík R., Okuliarová M., Kováčová E., Zeman M. (2014). Diquat-induced cytotoxicity on Vero and HeLa cell lines: Effect of melatonin and dihydromelatonin. Interdiscip. Toxicol..

[4] Mehmandost N., García-Valverde M.T., Laura Soriano M., Goudarzi N., Lucena R., Chamjangali M.A., Cardenas S. (2020). Heracleum persicum based biosorbent for the removal of paraquat and diquat from waters. J. Environ. Chem. Eng..

[5] Conde-Cid M., Paradelo R., Fernández-Calviño D., Pérez-Novo C., Nóvoa-Múñoz J.C., Arias-Estévez M. (2017). Retention of quaternary ammonium herbicides by acid vineyard soils with different organic matter and Cu contents. Geoderma.

[6] López-Blanco R., Moreno-González D., Nortes-Méndez R., García-Reyes J.F., Molina-Díaz A., Gilbert-López B. (2018). Experimental and theoretical determination of pesticide processing factors to model their behavior during virgin olive oil production. Food Chem..

[7] Ortiz G.G., Pacheco-Moisés F.P., Mireles-Ramírez M.A., Flores-Alvarado L.J., González-Usigli H., Sánchez-López A.L., Sánchez-Romero L., Velázquez-Brizuela I.E., González-Renovato E.D., Torres-Sánchez E.D. (2016). Oxidative Stress and Parkinson’s Disease: Effects on Environmental Toxicology. Free Radicals and Diseases.

[8] Fortenberry G.Z., Beckman J., Schwartz A., Prado J.B., Graham L.S., Higgins S., Lackovic M., Mulay P., Bojes H., Waltz J. (2016). Magnitude and characteristics of acute paraquat- and diquat-related illnesses in the US: 1998–2013. Environ. Res..

[9] Sha O., Cui B., Chen X., Liu H., Yao J., Zhu Y. (2020). Separation and determination of paraquat and diquat in human plasma and urine by magnetic dispersive solid phase extraction coupled with high-performance liquid chromatography. J. Anal. Methods Chem..

[10] Pan M., Xiang P., Yu Z., Zhao Y., Yan H. (2019). Development of a high-throughput screening analysis for 288 drugs and poisons in human blood using Orbitrap technology with gas chromatography-high resolution accurate mass spectrometry. J. Chromatogr. A.

[11] Yastrub T.O., Omelchuk S.T., Yastrub A.M. (2020). Dermal absorption of diquat and potential occupational risk. Wiad. Lek..

[12] McCuaig L.M., Martyniuk C.J., Marlatt V.L. (2020). Morphometric and proteomic responses of early-life stage rainbow trout (Oncorhynchus mykiss) to the aquatic herbicide diquat dibromide. Aquat. Toxicol..

[13] Tajai P., Suriyo T., Rangkadilok N., Fedeles B.I., Essigmann J.M., Satayavivad J. (2020). Andrographolide, an antioxidant, counteracts paraquatinduced mutagenesis in mammalian cells. Asian Pac. J. Cancer Prev..

[14] Soares J.J., Rodrigues D.T., Gonçalves M.B., Lemos M.C., Gallarreta M.S., Bianchini M.C., Gayer M.C., Puntel R.L., Roehrs R., Denardin E.L.G. (2017). Paraquat exposure-induced Parkinson’s disease-like symptoms and oxidative stress in Drosophila melanogaster: Neuroprotective effect of Bougainvillea glabra Choisy. Biomed. Pharmacother..

[15] Zhou Y., Lu M., Weng J., Wang Z., Sun F., Geng P., Wang S., Hu L., Gao Z., Wang X. (2017). Serum metabolic changes in rats of acute paraquat poisoning treated by pirfenidone. Int. J. Clin. Exp. Med..

[16] Cao S., Shen Z., Wang C., Zhang Q., Hong Q., He Y., Hu C. (2019). Resveratrol improves intestinal barrier function, alleviates mitochondrial dysfunction and induces mitophagy in diquat challenged piglets. Food Funct..

[17] Llabres-Campaner P.J., Guijarro L., Giarratano C., Ballesteros-Garrido R., Zaragozá R.J., Aurell M.J., García-España E., Ballesteros R., Abarca B. (2017). Synthesis, optical properties, and DNA interaction of new diquats based on triazolopyridines and triazoloquinolines. Chem.—A Eur. J..

[18] Ortiz G.G., Reiter R.J., Zúñiga G., Melchiorri D., Sewerynek E., Pablos M.I., Oh C.S., García J.J., Bitzer-Quintero O.K. (2000). Genotoxicity of paraquat: Micronuclei induced in bone marrow and peripheral blood are inhibited by melatonin. Mutat. Res.—Genet. Toxicol. Environ. Mutagen..

[19] Muangphra P., Kwankua W., Gooneratne R. (2014). Genotoxic effects of glyphosate or paraquat on earthworm coelomocytes. Environ. Toxicol..

[20] Kim S.J., Gil H.W., Yang J.O., Lee E.Y., Hong S.Y. (2009). The clinical features of acute kidney injury in patients with acute paraquat intoxication. Nephrol. Dial. Transplant..

[21] Mandal S., Pathak M.P., Sharma Bora N., Patowary P., Barman P.K., Kishor S., Goyary D., Verma N., Chattopadhyay P. (2019). Determination of LCt50 of aerosolized paraquat and its pulmonary toxic implications in non-anesthetized rats. Drug. Chem. Toxicol..

[22] Adam A., Smith L.L., Cohen G.M. (1990). An evaluation of the redox cycling potencies of paraquat and nitrofurantoin in microsomal and lung slice systems. Biochem. Pharmacol..

[23] Chowdhury A.R., Zielonka J., Kalyanaraman B., Hartley R.C., Murphy M.P., Avadhani N.G. (2020). Mitochondria-targeted paraquat and metformin mediate ROS production to induce multiple pathways of retrograde signaling: A dose-dependent phenomenon. Redox Biol..

[24] Zhan Y., Guo Z., Zheng F., Zhang Z., Li K., Wang Q., Wang L., Cai Z., Chen N., Wu S. (2020). Reactive oxygen species regulate miR-17-5p expression via DNA methylation in paraquat-induced nerve cell damage. Environ. Toxicol..

[25] Jalil A.S., Reddy S.B., Plautz C.Z. (2019). Cellular effects of diquat dibromide exposure: Interference with Wnt signaling and cytoskeletal development. Toxicol. Res. Appl..

[26] Magalhães N., Carvalho F., Dinis-Oliveira R.J. (2018). Human and experimental toxicology of diquat poisoning: Toxicokinetics, mechanisms of toxicity, clinical features, and treatment. Hum. Exp. Toxicol..

[27] Babalola O.O., Truter J.C., Van Wyk J.H. (2020). Lethal and Teratogenic Impacts of imazapyr, diquat dibromide, and glufosinate ammonium herbicide formulations using frog embryo teratogenesis assay-xenopus (FETAX). Arch. Environ. Contam. Toxicol..

[28] Vanholder R., Colardyn F., De Reuck J., Praet M., Lameire N., Ringoir S. (1981). Diquat intoxication: Report of two cases and review of the literature. Am. J. Med..

[29] Niu L.M., Liu Y., Lian K.Q., Ma L., Kang W.J. (2018). Characterization of a sensitive biosensor based on an unmodified DNA and gold nanoparticle composite and its application in diquat determination. Arab. J. Chem..

[30] Stobiecka M. (2013). Novel DNA-Biosensors for Studies of GMO, Pesticides and Herbicides. State of the Art in Biosensors—Environmental and Medical Applications.

[31] Dahiya V., Chaubey B., Dhaharwal A.K., Pal S. (2017). Solvent-dependent binding interactions of the organophosphate pesticide, chlorpyrifos (CPF), and its metabolite, 3,5,6-trichloro-2-pyridinol (TCPy), with Bovine Serum Albumin (BSA): A comparative fluorescence quenching analysis. Pestic. Biochem. Physiol..

[32] Silva D., Cortez C.M., Cunha-Bastos J., Louro S.R.W. (2004). Methyl parathion interaction with human and bovine serum albumin. Toxicol. Lett..

[33] Su C., Liu S., Cao S., Yin S., Zhou C., Gao S., Jia C., Ji Y., Liu Y. (2020). Self-assembled bovine serum albumin nanoparticles as pesticide delivery vectors for controlling trunk-boring pests. J. Nanobiotechnol..

[34] Jafari F., Moradi S., Nowroozi A., Sadrjavadi K., Hosseinzadeh L., Shahlaei M. (2017). Exploring the binding mechanism of paraquat to DNA by a combination of spectroscopic, cellular uptake, molecular docking and molecular dynamics simulation methods. New J. Chem..

[35] Gupta S., Kleiner H.E., Rogers L.K., Lau S.S., Smith C.V. (1997). Redox stress and hepatic DNA fragmentation induced by diquat in vivo are not accompanied by increased 8-hydroxydeoxyguanosine contents. Redox Rep..

[36] Zhang Q., Wang C., Liu W., Zhang X., Zhuang S. (2012). Evidence for DNA-diquat interaction and cytotoxicity in in vitro rat cells. Environ. Chem. Lett..

[37] Zhang G., Wang Y., Zhang H., Tang S., Tao W. (2007). Human serum albumin interaction with paraquat studied using spectroscopic methods. Pestic. Biochem. Physiol..

[38] Tunç S., Duman O., Soylu I., Kanci Bozoǧlan B. (2014). Study on the bindings of dichlorprop and diquat dibromide herbicides to human serum albumin by spectroscopic methods. J. Hazard. Mater..

[39] Moriwaki H., Yamada K., Nakanishi H. (2017). Evaluation of the Interaction between Pesticides and a Cell Membrane Model by Surface Plasmon Resonance Spectroscopy Analysis. J. Agric. Food Chem..

[40] Balleza D., Mescola A., Marín–Medina N., Ragazzini G., Pieruccini M., Facci P., Alessandrini A. (2019). Complex Phase Behavior of GUVs Containing Different Sphingomyelins. Biophys. J..

[41] Mescola A., Marín-Medina N., Ragazzini G., Accolla M., Alessandrini A. (2019). Magainin-H2 effects on the permeabilization and mechanical properties of giant unilamellar vesicles. J. Colloid Interface Sci..

[42] Kreutzberger M.A., Pokorny A., Almeida P.F. (2017). Daptomycin-phosphatidylglycerol domains in lipid membranes. Langmuir.

[43] Macii F., Salvadori G., Bonini R., Giannarelli S., Mennucci B., Biver T. (2019). Binding of model polycyclic aromatic hydrocarbons and carbamate-pesticides to DNA, BSA, micelles and liposomes. Spectrochim. Acta—Part A Mol. Biomol. Spectrosc..

[44] Biver T., Secco F., Tinè M.R., Venturini M., Bencini A., Bianchi A., Giorgi C. (2004). Intercalation of Zn(II) and Cu(II) complexes of the cyclic polyamine Neotrien into DNA: Equilibria and kinetics. J. Inorg. Biochem..

[45] Agonigi G., Biancalana L., Lupo M.G., Montopoli M., Ferri N., Zacchini S., Binacchi F., Biver T., Campanella B., Pampaloni G. (2020). Exploring the anticancer potential of diiron bis-cyclopentadienyl complexes with bridging hydrocarbyl ligands: Behavior in aqueous media and in vitro cytotoxicity. Organometallics.

[46] Aydinoglu S., Biver T., Secco F., Venturini M. (2014). Effects of micelle nature and concentration on the acid dissociation constants of the metal extractor PADA. Colloids Surfaces A Physicochem. Eng. Asp..

[47] Biancardi A., Biver T., Burgalassi A., Mattonai M., Secco F., Venturini M. (2014). Mechanistic aspects of thioflavin-T self-aggregation and DNA binding: Evidence for dimer attack on DNA grooves. Phys. Chem. Chem. Phys..

[48] Frisch M.J., Trucks G.W., Schlegel H.E., Scuseria G.E., Robb M.A., Cheeseman J.R., Scalmani G., Barone V., Petersson G.A., Nakatsuji H. (2016). Gaussian 16. Gaussian Inc. Wallingford CT.

[49] Cancès E., Mennucci B., Tomasi J. (1997). A new integral equation formalism for the polarizable continuum model: Theoretical background and applications to Isotropic and anisotropic dielectrics. J. Chem. Phys..

[50] Tomasi J., Mennucci B., Cammi R. (2005). Quantum mechanical continuum solvation models. Chem. Rev..

[51] Lepecq J.B., Paoletti C. (1967). A fluorescent complex between ethidium bromide and nucleic acids. Physical-Chemical characterization. J. Mol. Biol..

[52] Rocco D., Batchelor L.K., Agonigi G., Braccini S., Chiellini F., Schoch S., Biver T., Funaioli T., Zacchini S., Biancalana L. (2019). Anticancer potential of diiron vinyliminium complexes. Chem.—A Eur. J..

[53] Franklin R.E., Gosling R.G. (1953). The structure of sodium thymonucleate fibres. I. The influence of water content. Acta Crystallogr..

[54] Lakowicz J.R. (2006). Principles of Fluorescence Spectroscopy.

[55] Macii F., Biver T. (2021). Spectrofluorimetric analysis of the binding of a target molecule to serum albumin: Tricky aspects and tips. J. Inorg. Biochem..

[56] Tayeh N., Rungassamy T., Albani J.R. (2009). Fluorescence spectral resolution of tryptophan residues in bovine and human serum albumins. J. Pharm. Biomed. Anal..

[57] Khrapunov S. (2018). The enthalpy-entropy compensation phenomenon. limitations for the use of some basic thermodynamic equations. Curr. Protein Pept. Sci..

[58] Ryde U. (2014). A fundamental view of enthalpy-entropy compensation. Medchemcomm.

[59] Dragan A.I., Read C.M., Crane-Robinson C. (2017). Enthalpy–entropy compensation: The role of solvation. Eur. Biophys. J..

[60] Kenji W., Mizutani T., Hideki M., Susumu K. (2003). A new strategy for the design of water-soluble synthetic receptors: Specific recognition of DNA intercalators and diamines. Chem.—A Eur. J..

[61] Sudlow G., Birkett D.J., Wade D.N. (1975). The characterization of two specific drug binding sites on human serum albumin. Mol. Pharmacol..

[62] Platford R.F. (1983). The octanol-water partitioning of some hydrophobic and hydrophilic compounds. Chemosphere.

[63] Tomlin C.D.S. (2003). The Pesticide Manual.

[64] Olson R.E., Christ D.D. (1996). Chapter 33. Plasma protein binding of drugs. Annu. Rep. Med. Chem..

[65] Gou Y., Yang F., Liang H. (2016). Designing prodrugs based on special residues of human serum albumin. Curr. Top. Med. Chem..

[66] Topală T., Bodoki A., Oprean L., Oprean R. (2014). Bovine serum albumin interactions with metal complexes. Clujul Med..

