# Supplementary material for: Spectroscopic Analysis of the Binding of Paraquat and Diquat Herbicides to Biosubstrates
Source: Int J Environ Res Public Health. 2021 Mar 2;18(5):2412. doi: 10.3390/ijerph18052412 (PMC7967551; doi:10.3390/ijerph18052412)
Supplement: Supplementary file 1 [file ijerph-18-02412-s001.pdf]

Supporting Information

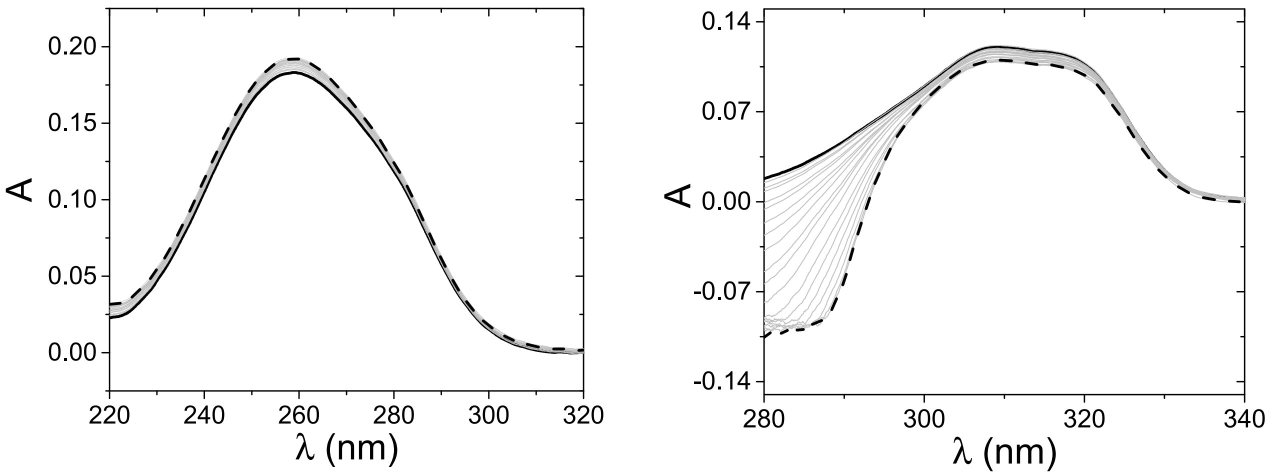

(**a**) (**b**)

Figure S1. Spectrophotometric titrations of (a) PQ/DNA (C_{PQ} = 9.30×10^{-6} M, C_{DNA} from 0 (solid) to 6.49×10^{-4} M (dash)) and (B) DQ/DNA (C_{DQ} = 6.87×10^{-6} M, C_{DNA} from 0 (solid) to 7.71×10^{-6} M (dash)). NaCl 0.1 M, NaCac 2.5 mM, pH 7.0, 37.0°C.

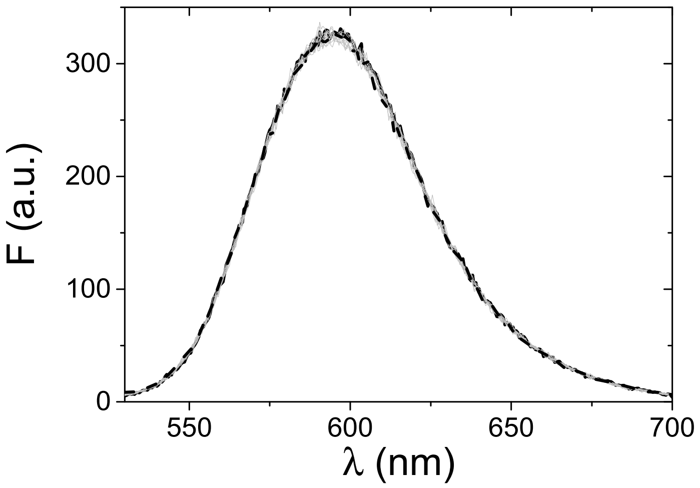

Figure S2. Fluorescence exchange titration of EtBr-saturated DNA with DQ at 25.0°C (C_{EtBr} = 9.96×10^{-5} M, C_{DNA} = 2.43×10^{-4} M, C_{DQ} from 0 (solid) to 8.03×10^{-4} M (dash), NaCl 0.1 M, NaCac 2.5 mM, pH 7.0, 25.0°C, λ_{exc} = 510 nm, λ_{em} = 595 nm.

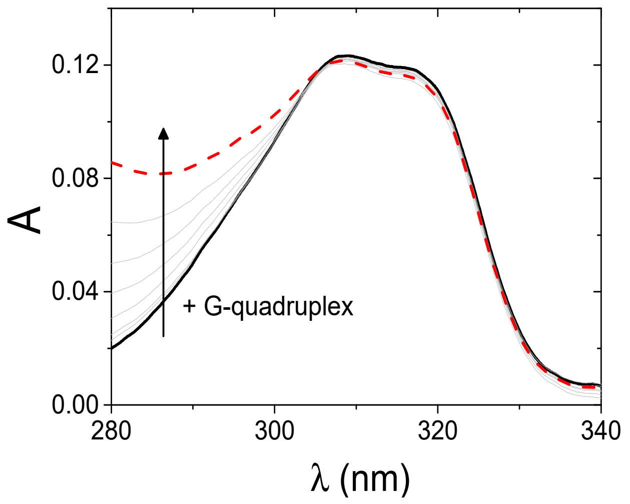

Figure S3. Spectrophotometric titration for the Tel23-DQ system; C_{Tel} = 7.77×10^{-9} (—) – 5.00×10^{-7} M (− −) ; C_{DQ} = 8.87×10^{-6} M, KCl 0.1 M, KCac 2.5 mM, pH 7.0, 25.0 °C.

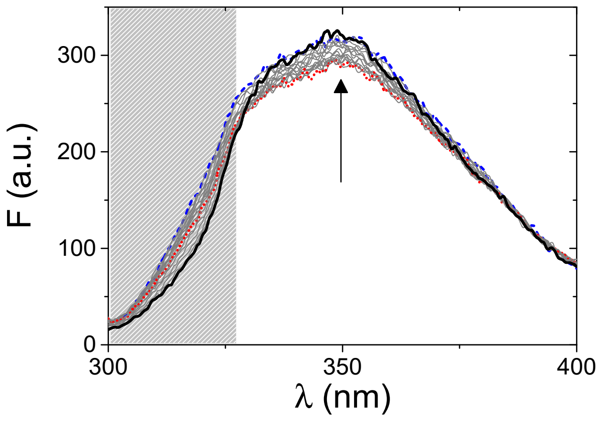

Figure S4. Spectrofluorimetric titration for the DQ/BSA system. The grey area highlights the distortions due to DQ absorbance effects. The arrow indicates the fluorescence emission profile likely due to free DQ contribution, which becomes evident for C_{DQ} > 1.86×10^{-6} M; accordingly, the binding isotherm in Figure 7 is cut at C_{DQ} < 1.86×10^{-6} M; C_{BSA} = 1.50×10^{-6} M, C_{DQ} from 0 (dashed blue line) to 1.86×10^{-6} M (dotted red line) and 1.07×10^{-5} M (straight black line), NaCl 0.1 M, NaCac 2.5 mM, pH 7.0, 37.0°C, λ_{exc} = 295 nm.

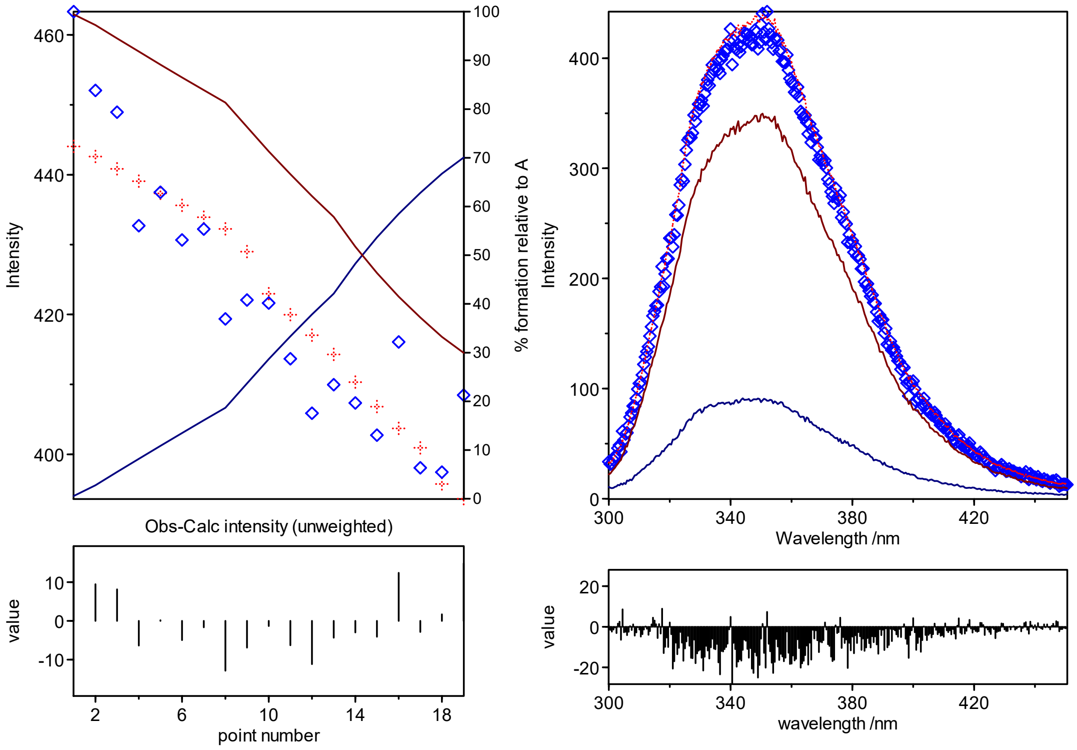

Figure S5. HypSpec2014 analysis of the fluorescence changes observed upon addition of DQ to BSA; C_{BSA} = 1.50×10^{-6} M, C_{DQ} from 0 to 1.77×10^{-6} M, NaCl 0.1 M, NaCac 2.5 mM, pH 7.0, 37.0°C, λ_{exc} = 295 nm. Left: titration curve at 345 nm (open diamond = experimental, cross = calculated) and species distribution (dark red = free BSA, red = PQ/BSA adduct). Right: fluorescence spectrum ((open diamond = experimental, dashed red line = calculated) and relevant deconvolution (dark red = free BSA, red = PQ/BSA adduct). The bottom panels are the residuals.

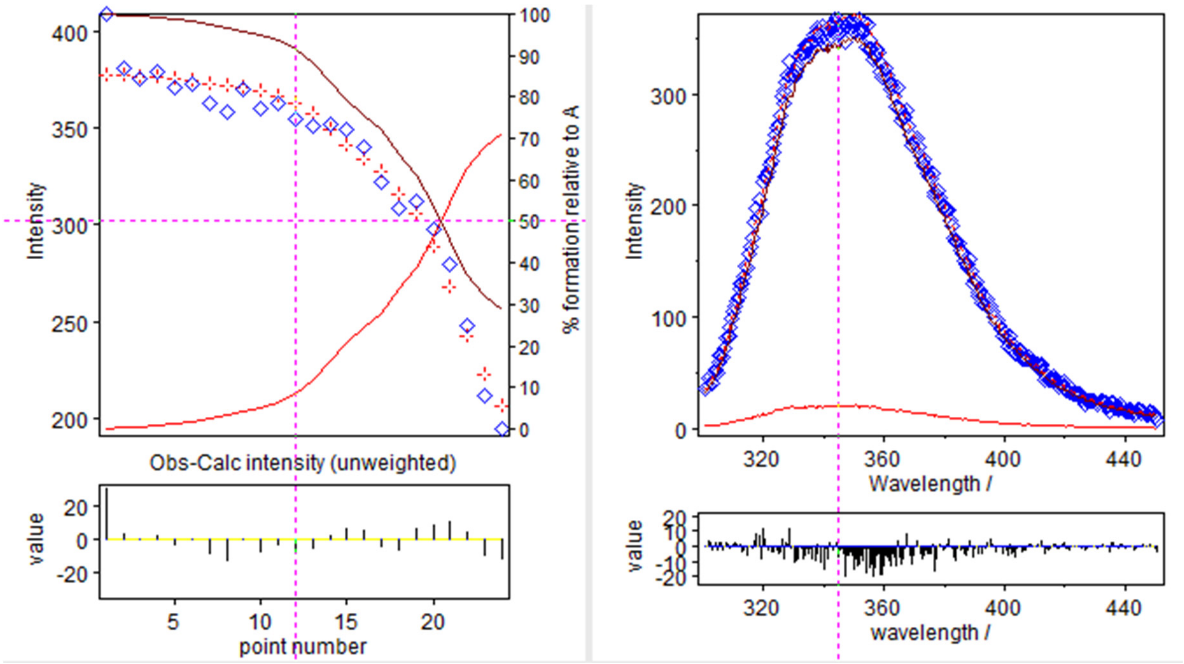

Figure S6. HypSpec2014 analysis of the fluorescence changes observed upon addition of PQ to BSA; C_{BSA} = 1.54×10^{-6} M, C_{PQ} from 0 to 5.12×10^{-5} M, NaCl 0.1 M, NaCac 2.5 mM, pH 7.0, 37.0°C, λ_{exc} = 295 nm. Left: titration curve at 345 nm (open diamond = experimental, cross = calculated) and species distribution (dark red = free BSA, red = PQ/BSA adduct). Right: fluorescence spectrum ((open diamond = experimental, dashed red line = calculated) and relevant deconvolution (dark red = free BSA, red = PQ/BSA adduct). The bottom panels are the residuals.

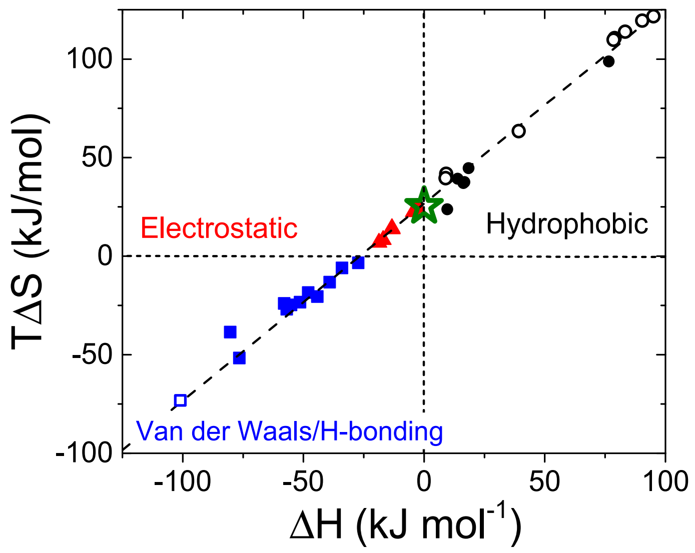

Figure S7. Plot of ΔH vs. TΔS for different ligands binding to BSA according to https://doi.org/10.1016/j.jinorgbio.2020.111305. Different points relate to the different driving forces for binding: (●) = hydrophobic forces, (■) = van der Waals/H-bonding, (▲) = electrostatic. Full point refer to organic molecules, open points refer to metal complexes. The star refers to what was found in this work for the PQ/BSA system.

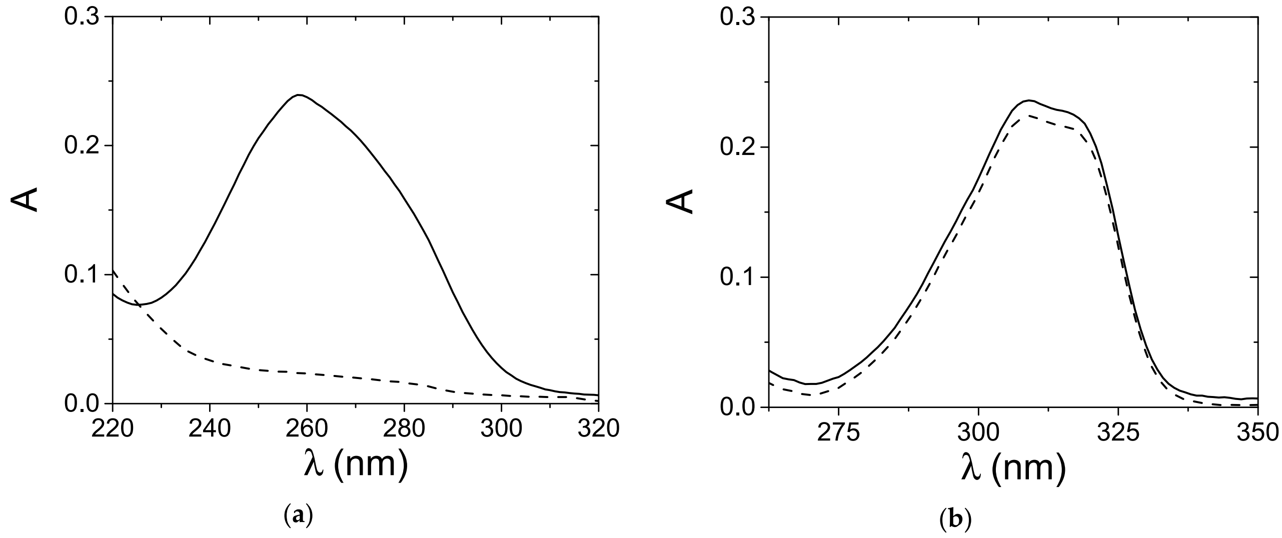

Figure S8. Absorbance spectra of (a) PQ in SDS 0.01 M before (solid) and after (dash) ultrafiltration and (b) DQ in DTAC 0.01 M before (solid) and after (dash) ultrafiltration; NaCl 0.1 M, NaCac 2.5 mM, pH 7.0.
